# Tracking Cancer Genetic Evolution using OncoTrack

**DOI:** 10.1038/srep29647

**Published:** 2016-07-14

**Authors:** Asoke K. Talukder, Mahima Agarwal, Kenneth H. Buetow, Patrice P. Denèfle

**Affiliations:** 1 InterpretOmics, Bangalore, India; 2 Computational Sciences and Informatics, CAS, Arizona State University, Tempe, USA; 3 Institut ROCHE, Boulogne, France

## Abstract

It is difficult for existing methods to quantify, and track the constant evolution of cancers due to high heterogeneity of mutations. However, structural variations associated with nucleotide number changes show repeatable patterns in localized regions of the genome. Here we introduce SPKMG, which generalizes nucleotide number based properties of genes, in statistical terms, at the genome-wide scale. It is measured from the normalized amount of aligned NGS reads in exonic regions of a gene. SPKMG values are calculated within OncoTrack. SPKMG values being continuous numeric variables provide a statistical metric to track DNA level changes. We show that SPKMG measures of cancer DNA show a normative pattern at the genome-wide scale. The analysis leads to the discovery of core cancer genes and also provides novel dynamic insights into the stage of cancer, including cancer development, progression, and metastasis. This technique will allow exome data to also be used for quantitative LOH/CNV analysis for tracking tumour progression and evolution with a higher efficiency.

Precision medicine is based on the concept of treatments tailored to patients’ molecular profiles^1^. SNPs (Single Nucleotide Polymorphisms) are the most frequent genetic variations in humans. For some monogenic diseases, some SNPs are directly linked to the cause^2^ of the disease; whereas, for many polygenic diseases, SNPs may eventually contribute to an often complex signature linked to the disease phenotype. With the goal of advancing precision medicine for cancer, most research has been focused on identifying SNPs for the stratification of patients at baseline and now more regularly when the patient disease relapses. The SNP profile in cancer is however extremely diverse, displaying inter-patient, intra-tumour, inter-metastatic, and intra-metastatic heterogeneity^3^, and is further compounded by genome mosaicism^4^. This mutational heterogeneity in tumours makes it difficult to develop therapies which will benefit many patients^5^. Additionally, one of the major challenges in anti-cancer therapeutics is the evolution of tumours to develop new mutations, making the previous treatments ineffective^6^. In addition, the rapid development of novel cancer immunotherapies is calling for a more holistic approach to neo-antigen biomarker and mutational load analysis than targeted sequence analysis^7^,^8^. Therefore, if robust, reproducible patterns, based on tumour evolution, can be identified within patient populations, this would propel the efforts for developing targeted therapies and precision medicine in cancer, benefiting a larger group.

In addition to SNPs, CNVs (Copy Number Variations) and LOH (Loss of Heterozygosity) are present in the genome. In CNVs, portions of the genome are deleted, amplified, translocated (intra-chromosome or inter-chromosome), or inverted^9^. Theoretically the number of copies of a section of DNA throughout the human genome will be 2 (bi-allelic). But in reality, the number of copies of a localized genomic region could in fact be 0, 1, 2, 3, 4 or more (any integer value). Copy numbers 0 and 1 represent deletion and LOH respectively. Copy numbers 3 and higher integers signify amplification. Thiagalingam *et al*. in their paper on LOH found that each of the five chromosomes analysed was affected by LOH with high frequencies, ranging from 47% to 78%^10^. The experiment showed that there are repeatable patterns in LOH in regions within DNA. Ni *et al*., showed that CNVs also exhibit localized reproducible patterns in cancer^11^. There is also evidence for the heritability of some localized CNVs and LOHs in familial tumours^12^.

While LOHs/CNVs show some patters at a population level, the phenotypic associations of CNVs, particularly with respect to disease association, are not trivial, and are not well understood. Some studies failed to identify substantial association of overall CNV burden or specific CNVs with disease in genome-wide association studies^13^,^14^. On the other hand, others have found higher overall CNV burden in tumour samples compared to healthy^15^,^16^. While one study^14^ concluded that common CNVs are unlikely to contribute much to disease heritability for many diseases, another found that the proportion of rare CNVs was higher in the tumour samples compared to healthy samples^17^. Additionally, despite the uncertainty in association patterns, locus specific associations with CNVs have been described for many diseases, including cancer. For example, homozygous deletion of GSTM1 is found to be correlated with cancer prognosis^18^.

SNPs are binary, categorical, and heterogeneous. CNVs, when looked at as absolute sequence alterations are categorical and heterogeneous too. The boundaries (breakpoints) of CNVs can vary over a population, making phenotypic association difficult as ‘consensus’ CNV loci need to be identified^19^. While disease association of CNVs may be directly associated with change in copy number (as in the case of deletions or amplifications), it is more related to the length of the CNV and the number of genes covered^20^. This feature of CNVs implies that it may be more useful to study phenotypic associations at localized gene level, rather than for the large genomic region, where one CNV may span genic, multi-genic, and non-genic regions. Localized CNV polymorphisms have been associated with many cancers^21^,^22^. There is also evidence that localized CNVs have an impact on pharmacodynamics due to altered metabolism of compounds^23^. Such CNVs affect the efficacy and toxicity of drugs through regulation of proteins involved in metabolism^23^. Some CNVs have been associated with high drug toxicity and increased incidence of adverse events due to increase in copy number^23^.

Although all this evidence indicates that LOHs/CNVs have inherent characteristics specific to a disease, there is insufficient evidence to conclusively define locus-disease associations. Since CNVs generally result in a change in nucleotide number at specific locations, this presents an opportunity for the quantification of these DNA level variations. Though, this will exclude the regions with copy number neutral variations. We hypothesized that the disease-specific concordance for localized CNVs is a disease characteristic. We therefore hypothesized that nucleotide level loss (or gain) in the coding regions of DNA with copy number 0, 1, 3, …, N, will show a cancer specific homogeneous statistical pattern. Moreover, this gene specific measure will have phenotype association. In other words, our hypothesis generalizes the principle of localized LOHs/CNVs for cancer, through the quantification of DNA, based on the change in nucleotide number (loss or gain) in the coding regions of the DNA.

DNA pileup is a technique that was originally used by SAMtools^24^ for measuring the depth of coverage of NGS data, and is used in many tools such as, GATK^25^, XHMM^26^ etc. We explored the concept of pileup in the context of LOHs/CNVs at a quantitative level, but with a different approach. Any LOH, CNV or structural variation in an exon, be it a deletion, amplification, or translocation, will have a quantitative effect on the protein product. The nucleotide count within the exonic regions of a gene will decrease in case of a deletion, and increase in case of an amplification. In the case of translocation, it will decrease at some gene and increase at some other.

Using an adaptation of the pileup concept, we quantified DNA content variation within a gene at the genome-wide scale and named this SPKMG. We formulated SPKMG (Sequence Per Kilobase of exon, per Megabase of the mappable Genome) measures for each gene. Unlike mutations, which are binary, the SPKMG measure for a gene is a real number – i.e., both numeric and continuous. A very high SPKMG value signifies amplification of a portion of a gene; a low SPKMG value signifies deletion of a part of the gene; whereas, zero SPKMG means the gene has been entirely deleted. Since SPKMG values are real numbers, we will be able to employ statistical techniques to unleash the structure and functional relationship of these numbers for a population. SPKMG values are calculated within a software which we have named OncoTrack.

We hypothesized that if nucleotide count alterations due to structural variations in the genome are truly associated with a tumour state, then we will be able to capture these and find some meaningful phenotypic associations through SPKMG. For this, we looked at the SPKMG measure from two perspectives, case/control comparative analysis, and control-free analyses using correlation and mutual information. Our goal was to assess the usefulness of SPKMG as an improved way of looking for population level patterns for tracking and predicting molecular changes in tumours. In this paper we explore the efficacy of SPKMG as a more efficient quantification technique for precision medicine and for tracking cancer progression.

To establish our theory, we have used total 69 exome datasets that comprise of 11 non-BRCA1/2 familial breast cancer (BC)^27^, 19 Esophageal Squamous Cell Carcinoma (ESCC) tumour samples (T) with 19 ESCC matching germline normal (N) samples^28^, and 20 healthy (control) samples (H)^27^,^29^. The OncoTrack analysis pipeline architecture is shown in Fig. 1.

## Results

### Non-BRCA1/BRCA2 Familial Breast Cancer Population

The SPKMG was computed for the breast cancer patients’ data. These are given in Supplementary Table 1. We compared the SPKMG values for the 11 breast cancer patients with the reference population of 20 healthy data (Supplementary Table 2). Figure 2(a,b) show the MDS plot, constructed using the edgeR^30^,^31^ MDS algorithm, and hierarchical clustering for SPKMG for all 31 samples. It is seen that the breast cancer patients are clustering closely compared to healthy patients (Fig. 2). This is in line with Thiagalingam *et al*.^10^ and Ni *et al*.^11^ observations that there are reproducible patterns in LOHs and CNV across the cancer patients. This shows that SPKMG follows a quantitative and normative pattern that has the potential to be used to define quantitative properties of a cancer population. To further examine the properties of SPKMG, we compared the values between the healthy samples and breast cancer patients, as well as within the breast cancer patients.

### Breast Cancer Comparative Analysis (Case/Control)

We performed the comparative analysis of the breast cancer population and the control population (Supplementary Table 2) using edgeR. We selected the most statistically significant genes with p-values ≤ 1.0e-20 (or adjusted p-values ≤ 1.0e-17). This gave us 28 genes with adjusted p-values ranging from 6.3E-063 to 5.5E-018 (Table 1). An analysis of the COSMIC^32^ database showed that all these 28 genes have been discovered in multiple cancer studies, with study counts ranging from 6 for KIR3DL1 to 43 for FLG. All these genes have been widely researched with the number of PubMed citations ranging from 8 for SPRRD2 to 92 for FLG (in the References section of COSMIC database). We also performed a manual bibliomic analysis to verify the role of these 28 genes in cancer progression; the results are listed in Supplementary Information 1. We hypothesize that these genes are the driver genes for breast cancer, because, all these genes are derived from DNA data and are common across multiple cancers.

#### Enriched Pathways

From the results of the comparative analysis between cancer and healthy populations, we selected the genes with adjusted p-values ≤ 1.0e-5. 1106 genes were significant following this criteria. Out of these 1106 genes, 146 genes are part of the 168 KEGG cancer and metabolism related pathways^33^. The pathway enrichment analysis results from XomPathways^34^ are in Supplementary Table 3. Seven pathways with adjusted p-values (Benjamini-Hochberg) less than 0.05 were obtained. These are, ‘Antigen processing and presentation’, ‘Natural killer cell mediated cytotoxicity’, ‘Androgen and estrogen metabolism’, ‘Custom calcium channel’, ‘Toll-like receptor signalling pathway’, ‘Pentose and glucuronate interconversions’, ‘Custom calcium channel A1’. These pathways formed three individual clusters, associated with the immune response and initiation of tumours, representing oncogenesis and associated inflammation^35^, calcium channels involved in proliferation and differentiation^36^, and metabolic pathways known to be associated with breast cancer^37^. These results show that the predisposition to breast cancer can be picked up from the peripheral blood of the familial breast cancer patients. The most central pathway and gene in the gene-pathway interaction network were the ‘Phosphatidylinositol signalling system’ pathway and the gene ITPR1. There is much evidence to support the role of the ‘phosphatidylinositol signalling pathway’ in cancer and its role in insensitivity to calorie restriction^38^,^39^. Figure 3 shows the pathway-gene, gene-gene, and pathway-pathway graphs for the case/control significant genes and their respective pathways.

### Breast Cancer Control-free Analysis using Mutual Information

The breast cancer control-free Mutual Information (MI) network was constructed by using 0.05 as threshold for removing the weakest edge of each triple of nodes in parmigene (using ARACNe)^40^,^41^ and removing all gene-pairs with MI coefficients less than 1. This gave us 1405 genes with high MI. We then did graph theoretic analysis using igraph package^42^. The MI network of the breast cancer population is shown in Fig. 4(a). We selected the top 18 genes that had highest degrees (8 and above). All these genes are responsible for multiple carcinomas. These genes are PPP1R15B (30 tissues), TTC21B (35 tissues), XIRP2 (47 tissues), KIAA1143 (16 tissues), BCHE (36 tissues), GLRB (33 tissues), JAKMIP2 (40 tissues), OR13C8 (19 tissues), ZNF790 (29 tissues), POF1B (38 tissues), ARHGAP24 (26 tissues), DACH1 (40tissues), LIPI (38 tissues), SPAM1 (34 tissues), DIAPH3 (38 tissues), CSMD3 (49 tissues), DCC (44 tissues), and FLRT3 (22 tissues). The tissue numbers are taken from the COSMIC database.

We then looked at the properties of the breast cancer MI network and how it compares with other biological networks. The MI network of 1405 genes had 1172 edges. The network had a diameter of 13 and a clustering coefficient of 0.446. Metabolic networks have a clustering coefficient of 0.67^43^. The degree distribution, P(k), gives the probability that a randomly selected node will have *k* links. We found that the MI network followed a power law degree distribution, P(k)~k^−α^ as shown in Fig. 4(c) with an α (Alpha) of 2.44. Typically the value of α is 2 < α < 3 with occasional outliers for natural scale-free networks. Biological networks such as protein interaction networks and metabolic networks have similar values of alpha (2.4, and 2.2, respectively)^43^,^44^. The scale free nature of networks is expected to make them more resilient to general attacks. Such networks are less vulnerable to random node removal^45^.The largest degree for the MI network was 12 while the average degree was 1.67. Average path length of the MI network was 5.12; whereas, the average shortest path length for protein interaction network is 6.8, and, it is 2.56 for metabolic networks^43^. The network centralization was 0.0074, implying deviation from a star like topology. The degree based assortativity of the MI network was 0.638, implying that the network was assortative in nature. Assortativity of a network is measured from the correlation in degrees of pairs of connecting nodes^46^. In an assortative network, hubs tend to connect to other hubs in the network, whereas in disassortative networks, hubs connect to lower degree nodes. Biological networks have been found to display disassortativity, where high degree nodes (hubs) are more likely to connect to nodes of lower degree^46^,^47^. The assortativity of a network is associated with properties such as resilience and evolvability^46^,^48^. For gene regulatory networks, it has been observed that higher assortativity leads to higher robustness and lower evolvability of the network^48^,^49^. Whether this property extends to the MI network requires further investigation.

We also used the 1405 MI network genes in cluster analysis. We got a total of 474 clusters with gene memberships ranging from 2 to 119. We selected the clusters that have 10 or more members for further analysis. Figure 4(b) shows the subnetwork of genes from the clusters with 10 or more genes. We combined the resulting 9 clusters and top 19 highest degree genes to get 264 unique genes. We looked at the COSMIC database for these 264 genes. Out of these 264 genes, only 4 genes were not present in the COSMIC database as cancer genes. This implies that 98.5% of the genes that form clusters with high degree of connectedness within the MI network are already known to be associated with cancer.

We looked at the functional enrichment of these 264 genes in DAVID^50^,^51^. The enrichment analysis returned the terms ‘Cell-cell adhesion’, ‘Cell adhesion’ and ‘Biological adhesion’, with adjusted p-values less than 0.01 (Supplementary Information 2). These results indicate that the main modules in the network are associated with cell-cell adhesion. Further, we performed degree centrality, closeness centrality, betweenness centrality, and eigenvector centrality on the MI network, to obtain 28 unique genes (Supplementary Information 2). Out of these 28 genes, genes like DCC, DIAPH3, ARHGAP24, BCHE, DMD, LRP1B, BRWD3, DSC2, and HAS2 are known to be associated with metastasis and tumorigenesis (Supplementary Information 2).

We can see that the pathways that were significantly enriched in the case/control and control-free analyses are very different. However, the functions of these pathways tell a very interesting story. The case/control pathways, where we are comparing breast cancer samples with healthy data, are telling us that the pathways implicated are related to cancer initiation, growth and immune response, and even specifically an androgen and estrogen metabolism associated cancer. However, when we look at control-free MI results of breast cancer, we see enriched pathways that are mainly related to cell adhesion, with hub genes involved in metastasis. While the case-control analysis talks about the presence of cancer in the samples, the MI network genes and the implicated pathways are telling us that the cancer is displaying metastatic properties.

### Esophageal Squamous Cell Carcinoma

To understand the power of OncoTrack, we took another type of cancer data. In this case, we took 38 exome samples from the study^28^. These 38 samples contained tumour (with >70% malignant cells) and matched germline tissues (5 cm away from the tumour) from 19 esophageal squamous cell carcinoma (ESCC) patients. For this data, we examined the information that we obtained from the SNV and CNV analysis along with the SPKMG results.

### ESCC SNV and CNV Analysis

We used the GATK^23^ protocol for analysis of point mutations in the samples. We did this by considering both the tumour and matching normal tissue (germline) samples, unlike the original study^28^, where the focus was on identifying the somatic variants associated with cancer. Such a comparison enabled us to look at both the similarities as well as dissimilarities between the samples. After application of filtering criteria as described in Methods, we obtained between 567 to 1392 pathogenic variants across all samples. 20 mutations were common across all tumour samples, while 36 were common across the germline samples. All 20 mutations common in the tumour samples were also present in the germline samples. These are listed in Supplementary Information 3. When we looked at the mutations at the gene level, we found that 25 genes carrying the mutations were common across all 19 tumour samples. On the other hand, 39 genes were common across the 19 normal samples. All 25 genes which were common between the tumour samples were also common amongst the normal matching tissues. These genes are CDCP2, HRNR, GPATCH4, LOC375190, EDAR, TTN, ZNF717, SCAMP1, SMAD5, CYFIP2, PRIM2, SRRM3, EMID2, PRKDC, ZNF518A, TMEM216, DIXDC1, VPS11, EI24, PLCB2, C15orf57, SLC24A5, NPRL3, NLRC3, and CDC27.

We used XHMM^26^ protocol to identify the CNVs in the tumour and matching normal samples from the ESCC patients. The detailed analysis steps are given in Methods with the results in Supplementary Table 4. The minimum and maximum length of CNVs in tumour were 0.21 and 11989.11 Kbases respectively; whereas they were 0.12 and 12110.46 Kbases for matching normal tissues. The number of genes impacted by the CNVs ranged from 1 to 163 for tumour and 1 to 105 for normal tissues. We found a consistent pattern of more CNVs present in tumour compared to the normal samples. The average length (and total length) of the CNVs was also higher in the tumour samples. These results are consistent with some of the previous findings related to CNV burden^15^. While these common patterns were identified, there was little consistency across samples with respect to the genes present in the CNV regions. No genes were common across either the tumour samples or the matching normal samples. However, we did find varying levels of similarity between the tumour and matching tissue samples from the same patients. The number of common genes having CNV for this comparison ranged from 0 to 409. Therefore, we found an absence of gene specific consistent patterns across CNVs which limit its use for generalizability of characteristics and for cancer diagnosis and tracking.

### ESCC Comparative Analysis

We compared the SPKMG values across the healthy, tumour, and matched normal samples to get four different comparisons. We used edgeR to obtain the four sets of comparative analysis tables for healthy versus tumour comparison (represented as HxT), healthy versus matched normal comparison (represented as HxN), healthy samples versus all cancer patients samples, i.e. tumour and matched normal samples (represented as HxTN), and tumour versus matched normal samples (represented as NxT). The comparative analysis results for these are given in Supplementary Table 2.

We selected all genes with adjusted p-value < 1.0e-50 (Benjamini-Hochberg correction) from the HxT, HxN, and HxTN comparisons. With 1.0e-50 as the threshold for p-value, we got 598 genes in the HxT; 869 genes in HxN, and 897 genes in HxNT comparisons. A large overlap was seen in the genes as shown in Fig. 5. We selected all common genes from these 3 sets (adjusted p-value < 1.0e-50) and found 594 unique genes. Enrichment analysis for these genes using DAVID showed that they are overrepresented for pathways and processes related to Cadherin and Wnt signalling, and cell adhesion with adjusted p-values < 0.01. Therefore, when compared to healthy controls, both tumour and matched normal samples are similar and differ from healthy for these properties. The Cadherin mediated cell adhesion and Wnt signalling pathways together provide invasive and metastatic properties to cells and are involved in processes such as epithelial to mesenchymal transition^52^. We also observed that 62 out of the most significant 100 genes in the HxT comparison were common in the top most significant 100 genes in the HxN comparison. These genes were overrepresented for cell adhesion with an adjusted p-values of 0.0038. These results show large similarities between the tumour and matched normal samples in terms of CNVs which was not seen using the conventional CNV analysis.

For the NxT comparison, the genes with adjusted p-values less than 0.01 were identified. This returned 6 genes, all of which are located on chromosome 11q13. Amplification of this region (11q13) is of known significance in many cancers and is associated with metastasis^53^. Out of the 11 genes used for experimental validation of 11q13 amplification in oral squamous cell carcinoma^54^, 5 genes were present amongst the identified list of 6 significant genes. We found that these genes are overrepresented for the ‘Melanoma’ KEGG pathway with an adjusted p-value of 0.0038. Therefore, it appears that the main difference between the tumour and matching germline sets is amplification of 11q13 region containing cancer associated genes in the tumour samples.

### ESCC Control-free Analysis using Mutual Information

We performed MI network analysis of the ESCC tumour and germline populations with 19 samples. The weakest edge cut-off of each triple of nodes was set to 0.01, and MI coefficients greater than 1.4 were retained. Further, all genes with degree equal to 0 in the network were removed. We then studied the clustering of genes and clusters with 10 or more genes followed by functional analysis.

#### ESCC Tumour MI Network

The tumour MI network had 1520 genes with 1308 edges. The entire MI network for the tumour samples is given in Fig. 6(a). On isolation of key network clusters, the resulting graph contained 17 clusters and had 592 genes, and is shown in Fig. 6(b).

#### ESCC Germline MI Network

The germline MI network contained 2680 genes with 3597 edges (Fig. 7(a)). The subgraph formed from the identified gene clusters is shown in Fig. 7(b). This subgraph contained 1875 genes which formed 6 clusters. The functional annotation of this subnetwork was carried out using DAVID. These genes were significantly overrepresented for ‘Protein amino acid phosphorylation’ with adjusted p-value of 0.003. Amino acid phosphorylation plays a critical role in the activity of cellular enzymes and signal transduction.

### ESCC Control-free Analysis using Pearson Correlation

Mutual information provided us information about the relationship between genes, but it is neutral to the directionality of this relationship. To look at the interrelations along with the directionality, we used Pearson correlation (PC) to discover a different type of inherent relationship between the ESCC samples and genes. We first looked at how the germline and tumour samples correlate. We found that most samples show a correlation higher than 0.9 (Supplementary Table 5). The correlations were also calculated after removing the gene TTN, which had very high values across samples and could bias the results. The observed correlations remained very similar for most samples.

#### ESCC Pearson Correlation Network Link Analysis

We then studied the properties of the gene-gene correlation networks for the tumour and germline samples. The resulting complete germline PC network was much more interconnected compared to the complete tumour network. We did a link analysis for both the germline and tumour PC networks. We looked at the top 15 genes with highest degree of connectivity (links) in both the networks. The most linked genes in the tumour network were TAL1, HES2, FZD10, IER2, FRAT2, CLDN19, COL9A2, PHLDA1, LPPR2, TMEM125, KCNQ4, FAM19A5, ZBTB17, TTC22 and GSC2. Of these, genes such as FZD10, HES2 and COL9A2 with 2597, 2626 and 2572 degrees respectively are known to be involved in Cadherin, Wnt, and Integrin signalling pathways. Additionally, FZD10 promotes metastasis and promotes epithelial to mesenchymal transition through aberrant activation of WNT3-FZD10-β-catenin signalling^55^. IER2 is also known to promote tumour motility and metastasis, and is a potential prognostic and therapeutic target^56^. The most linked genes in the germline network were COL18A1, RNF26, CRB2, FZD10, NTN3, ACKR3, MRI1, NFATC1, HTR5A, SLC18A3, XKR8, NXPH4, WNT10A, DGKQ and ISLR2. Only FZD10 was common for both the networks. Of these, genes such as NFATC1, which is an oncogene^57^ and ACKR3 (6719 and 6721 degrees/links respectively), which is involved in tumour development and metastasis in many tumours^58^, are recognized as potential therapeutic targets. Other interesting genes include WNT10A, which promotes ESCC migration and invasion^59^ and RNF26 which is expected to be involved in carcinogenesis, but needs further investigation^60^.

### ESCC Germline/Tumour Control-free Network Properties

We further examined the properties of the PC and MI networks. The MI networks for both tumour and germline populations displayed scale free topology, with similar values of alpha (α_tumour_ = 2.54, α_germline_ = 2.52). The power law fits for both these networks are shown in Figs 6(c) and 7(c) for the tumour and germline networks respectively. The subnetworks with highly clustered genes for the tumour and germline samples contained 592 (17 clusters) and 1875 (6 clusters) genes respectively. Since the same filtering criteria were used for both germline/tumour networks, the difference in the number of genes and clusters indicates that the genes in the germline sub-network are more interconnected compared to the tumour. This pattern of more interconnections in the germline network were observed in the PC networks as well. The PC tumour network was more diffuse, with a diameter of 28, average path length of 5.6 and 0.6 clustering coefficient, compared to the germline network with 20 diameter, 3.37 average path length and a 0.8 clustering coefficient. Neither the germline, nor tumour complete PC networks displayed scale-free topology.

To track the changes in the network states, and to identify the key genes involved in the transformation from germline to tumour state, we looked at the network topology and the degrees (links) of connectivity of the genes in the PC networks. The overall correlation between the links in the germline and tumour correlation networks was 0.676. We then identified stretches of chromosomes (50 genes long) which showed large deviations from this value (Supplementary Table 6). We hypothesized that these regions would provide tracking information regarding the perturbation of the germline network and the transition to tumour state, and provide potential therapeutic targets. 4 regions with absolute correlation less than 0.3 were identified. These were located on chromosomes 3q13, 6q14-16, 8q22-24 and 11q13. Of these, regions such as 11q13 and 8q23-24 are known to be associated with cancers^53^,^61^. The regions showing very high correlations (>0.95) were on chromosomes 3q11-13, 10q25-26, 11p15 and large portions of chromosome X. Interestingly, while regions on 3q11-13 were highly correlated, the adjacent regions on 3q13 showed no correlation. Changes in neighbourhood for genes in such regions could be indicative of the shift from the germline to tumour state.

We also looked at the change in degrees of genes between the germline and tumour state. Following the hypothesis of the previous analysis, any genes displaying substantial alteration in their network behaviour and preferential attachment should be markers of the tumour development process, and can be potential therapeutic targets. The change in degrees from germline to tumour are given in Supplementary Table 7. The top 15 genes which showed a breakdown of preferential attachment and reduced from high degree genes in germline to tumour include genes such as SORBS2, ZHX2 which have tumour suppression activities^62^,^63^, whereas others such as EIF2S2, ZBTB14 and PPFIA1 promote tumour proliferation and invasion^64^,^65^,^66^. Of the top 6 genes with higher degrees in tumour network compared to germline, expression of KLK12 is known to be a diagnostic and prognostic marker of gastric cancer^67^.

We also found that for four genes significant in the NxT comparative analysis, there was a large loss in their connectivity (links) and preferential attachment in the tumour network compared to the germline network, as shown in Supplementary Table 7. This indicates that the changes in gene-gene interaction patterns of these genes are important in altering the state of the network and in the transition from the germline state to the tumour state. Therefore, these genes may be the driver genes for the transformation of the tissue. These results indicate that link count is a potential quantitative tracking biomarker which needs further investigation. We also looked at the negative and positive PC networks for the tumour and germline states. Both these networks showed similar trends as for the whole network, with higher density in germline compared to tumour state. Additionally, both the tumour and germline negative PC networks showed disassortative mixing patterns, with assortativity coefficients of −0.48 and −0.66 respectively. The positive PC networks on the other hand were more assortative in nature, with assortativity coefficients of 0.63 and 0.36 for the germline and tumour networks respectively.

## Discussion

Lack of combinatory tools and technologies, have for long, prohibited looking at DNA for quantitative molecular pathology. In this paper, we have presented a novel technique called SPKMG for quantitative DNA analysis. The SPKMG values are similar to RPKM values, applied in the exonic context. We have used various types of analytics to study properties of the SPKMG measure within OncoTrack software, and have found that the results from the different analyses show high levels of concordance. We have used cancer patients’ exome data to measure SPKMG and showed that it shows a normative pattern, and can be used as a tracking mechanism for a complex heterogeneous disease like cancer. This technique identifies common patterns across populations which can be used to define and track the phenotypic properties of cancer at a population level and provide interesting insights into the state of the cancer and cancer development. A comparison with CNV and SNV analysis showed the advantage of a continuous quantitative measure like SPKMG compared to categorical binary measures such as CNV calls and point mutations. While these measures are able to identify patterns at the individual patient level and show some characteristics at the overall level, specifically for CNV burden, they cannot be used for population level statistics which are useful in diagnosis and precision medicine.

The demonstration of the efficacy of SPKMG was further enhanced through the combination of case-control and control free analyses that provided a more comprehensive view of the tissue states. While the case-control analysis for breast cancer talked about the presence of cancer in the tissue, the control-free analysis was able to identify the presence of metastatic signals in the sample. This is particularly interesting considering the observation that in a high proportion of patients diagnosed with early stage breast cancer, the tumour has already metastasized^68^. The superior ability of OncoTrack to identify these states shows promise for providing a better metric for identifying the actual state of tumours. On the other hand, the case-control analysis for the ESCC tumour showed primary differences in cell adhesion and tumour development and metastasis related signals. Therefore using these analyses we were able to more accurately characterize the genetic state of the tumour. In addition, we were able to extract some interesting insights into the ESCC tumour states using the SPKMG values for the germline dataset.

OncoTrack was able to discover similarities and dissimilarities between tumour and the matching tissues based on their SPKMG values. The germline tissues from the ESCC patients were located 5 cm away from the surgical boundary, but presented many features similar to the tumour tissue. These included common significant cancer causing genes with the presence of similar cell adhesion alterations in the case-control comparison with healthy. This fits well with the theory of field cancerization where the cells surrounding the tumour also have similar characteristics due to experiencing the same conditions as the tumour tissue, but are non-cancerous^69^,^70^,^71^. Field cancerization is commonly seen in many epithelial cancers^72^. Additionally, the histologically normal tissue surrounding some tumours have been shown earlier to carry some signals of metastasis using FTIR spectroscopy^73^. Combining these theories, SPKMG helped us in concluding that the germline tissue surrounding the ESCC tumours in these samples is pre-malignant and is similar to the tissue which preceded the tumour. Then, by analysing the differences between the tumour and germline tissues, OncoTrack helped us identify the processes involved in the development of the tumour. Using tumour-germline comparisons, we were able to identify a chromosomal region whose amplification seems critical in the differentiation of the malignant state from the pre-malignant tissue. The identification of the same region in previous independent studies, using QPCR^54^ provides validation to the hypothesis and power of SPKMG. These results indicate that CNV information using SPKMG can be used to identify thresholds and prerequisites for malignant transformation, which were not observable using the traditional CNV calling algorithms. Since SPKMG can identify patterns in histologically normal pre-malignant tissue, it presents an opportunity for the prediction of cancer in tissues displaying tumour properties before the cancer develops, and for predicting cases with high likelihood of relapse. OncoTrack therefore displays immense potential for gathering wide-ranging information regarding the physical state of the DNA and the dynamics of tumour development and progression.

The greatest merit of SPKMG is that it will now enable DNA to be used for both mutation analysis and quantitative CNV/LOH analysis, therefore providing multiple levels of information from a single molecule. OncoTrack provides a powerful quantitative tool for tracking cancer states in the form of SPKMG, and may be combined with the various existing methods for mutation discovery from the exome. This makes DNA a very attractive molecule for precision medicine. The recent development of cancer immunotherapies addressing checkpoint inhibition^74^ calls for a novel way for defining tumour complexity and propensity to generate neoantigens at the genome level; whereas most current efforts are focused on candidate gene mutation analysis. In this context, it is interesting to note that many immune system related processes were identified as key functional alterations in the tumours, using OncoTrack, and can be further investigated to study how these processes may contribute to the patient’s response to different immunotherapies. A continuous variable monitoring method like SPKMG will provide valuable input for a more systemic tumour classification and pharmacology^23^. Additionally, this property of SPKMG will provide a tracking measure which can be used to predict future course of the disease and aid the development of appropriate treatment strategies. Moreover, SPKMG is not tissue specific unlike mRNA, but maintains the calibration property of mRNA expression, for the identification of response specific genetic patterns. Therefore, OncoTrack will help to understand diseases better from epidemiological and demographical point of view. The SPKMG marker therefore can be refined to offer a better, cheaper, faster, and more accurate tool for precision medicine and cancer screening for individual patients (n = 1) and for a population (n = N). We believe that SPKMG displays immense potential to serve as a single algorithm which can provide comprehensive information regarding the cancer genomic landscape and potential therapeutic targets, and will lay the foundation for further research that will help us in further modeling the dynamics of cancer development and metastasis toward a better cancer patients’ care.

We have tested the SPKMG algorithm only with cancer data; however, the phenomenon of LOH/CNV is universal. We therefore believe that SPKMG is not restricted to only cancer; and it will work equally well for neurological^75^ and other diseases where LOH/CNV associations are present. However, this demands further investigation.

## Methods

### Architecture of OncoTrack

**Figure 1:**
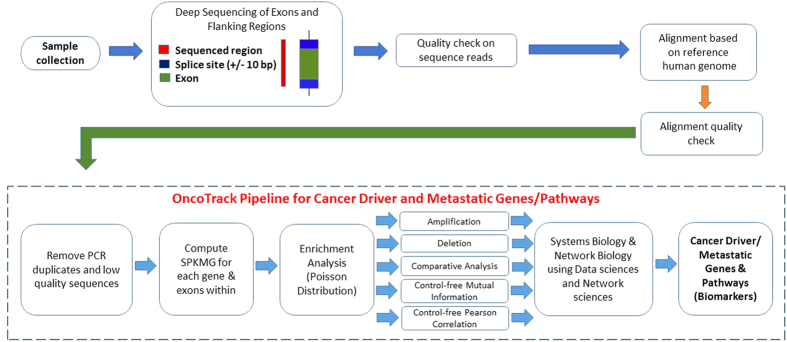
Architecture of OncoTrack pipeline.

**Figure 2:**
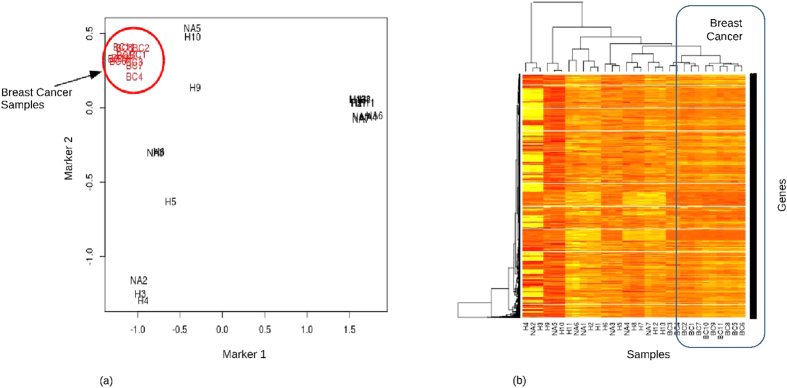
Clustering of Breast cancer and healthy individuals. (**a**) MDS plot of SPKMG values of 11 breast cancer and 20 healthy samples. Distances were calculated using log fold changes. Marker 1 and Marker 2 in this figure are the top two leading log fold change dimensions in the data. (**b**) SPKMG heatmap and hierarchical clustering of the breast cancer and healthy samples.

**Figure 3:**
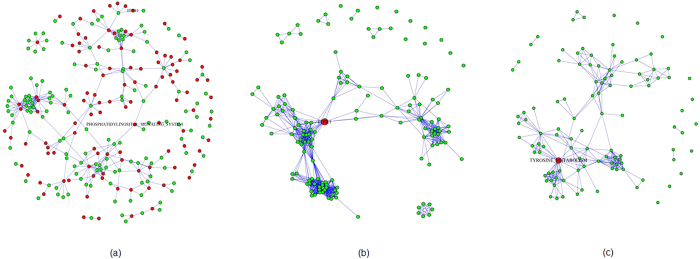
Enrichment networks from XomPathways for Breast cancer Comparative Analysis. (**a**) Enriched pathways – gene network. (**b**) Gene-gene network, with most central gene highlighted in red. (**c**) Pathway-pathway interaction network, with most central pathway in red.

**Figure 4:**
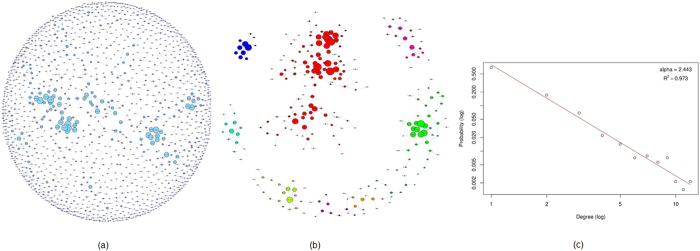
Breast cancer mutual information network. (**a**) The entire MI network for breast cancer samples consisting of 1405 genes and 1172 edges. (**b**) 9 clusters with 10 or more genes identified and isolated from the whole network. (**c**) The degree distribution follows power law with an alpha of 2.44.

**Figure 5:**
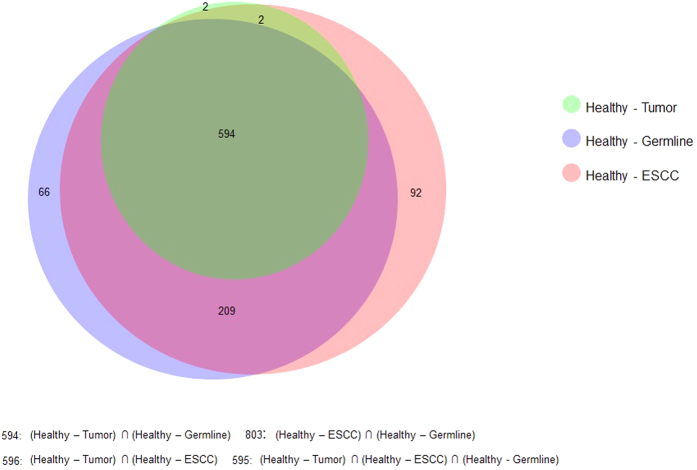
Overlap of significant genes from the comparative analyses for HxT, HxN and HxTN.

**Figure 6:**
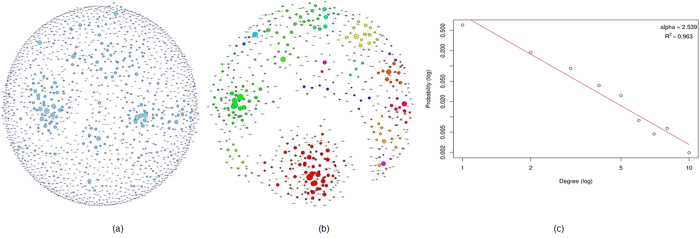
ESCC Mutual Information network for 19 tumor samples. (**a**) The entire MI network for ESCC tumor samples with 1520 genes and 1308 edges. (**b**) 17 clusters with 10 or more members identified and isolated from the complete network. (**c**) The degree distribution of the tumor MI network follows power law with an alpha of 2.54.

**Figure 7:**
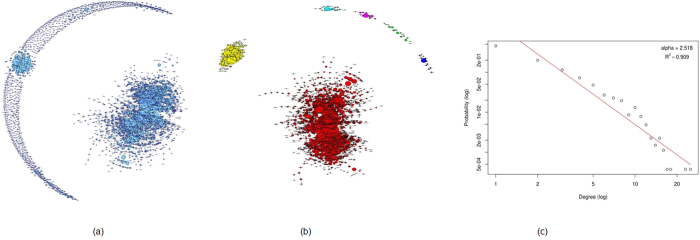
ESCC Mutual Information network for 19 germline samples. (**a**) The entire MI network for ESCC germline samples consisting of 2680 genes and 3597 edges. (**b**) 6 clusters with 10 or more genes identified and isolated from the MI network. (**c**) The degree distribution for the complete MI network follows power law with an alpha of 2.52.

The OncoTrack analysis pipeline architecture is shown in Fig. 1. We identified suitable publicly available exome datasets from SRA. We included datasets which were sequenced using Illumina HiSeq with the random Agilent sure-select library preparation protocol. This type of library preparation is required for being able to measure the SPKMG statistic. The SRA files were downloaded and converted to FASTQ format before further processing. The data were pre-processed, quality checked and cleaned with FastQC (http://www.bioinformatics.babraham.ac.uk/projects/fastqc), followed by alignment to hg19 reference genome (http://hgdownload.cse.ucsc.edu/downloads.html) using BowTie^76^ to create SAM/BAM files. This was followed by duplicate marking and indel-realignment, using Picard (http://picard.sourceforge.net) and GATK^25^ respectively. A collapsed geneset with exon coordinates was created from UCSC gene table for hg19 using technique described in XomAnnotate^34^ to obtain a single entry for a gene at a specific locus with all splice variations for that gene. This procedure collapsed the 44292 RefSeq rows of hg19 into 19358 genes. The BAM files were read using exonic location of individual genes in the genome as obtained by the collapsed gene step using BamTools API^77^. The BAM files were cleaned, i.e., PCR duplicates and low quality (Phred < 20) sequence reads were removed, and SPKMG measures were computed for the coding regions of each gene. Comparative analysis of the cancer population with respect to healthy was performed using edgeR^30^,^31^. We used statistical significance (adjusted p-values) to select the differential genes. These genes are used for bibliomic analysis, followed by functional enrichment analysis. We used Parmigene^40^ and ARACNe^41^ Mutual Information (MI) to create the MI network and shortlist core network genes. This was followed by systems biology and network analysis to examine the MI networks. Correlation networks were created using Pearson correlation with threshold of 0.95 absolute correlation, followed by network analysis and link analysis. Functional analysis on all shortlisted genes from various analyses above were performed using DAVID and 168 KEGG cancer and metabolic pathways using XomPathways. In addition to the SPKMG based analysis, we also carried out an analysis of CNVs and SNVs using the traditional SNV and CNV calling methods implemented in GATK^25^ and XHMM^26^.

### Data for this study

To test our model, we used exome data from studies on two types of cancers viz., breast cancer^27^ and esophageal squamous cell carcinoma (ESCC)^28^.

The first cancer dataset consists of exome data from 11 breast cancer patients with familial Non-BRCA1/BRCA2 breast cancer from France, Italy, the Netherlands, Australia, and Spain. The dataset comprises of patients from seven families having at least 6 breast cancer cases (between 6 and 10). None of the patients had familial BRCA1/BRCA2 pathogenic mutations and were diagnosed with breast cancer before the age of 60. No woman was affected with ovarian cancer in these families^27^.

The second cancer dataset is from Esophageal Squamous Cell Carcinoma (ESCC) patients with >70% malignant cells and matched germline tissues (adjacent esophageal epithelial tissue five centimetres away from the border of surgical area) collected from Cancer Institute/Hospital, Chinese Academy of Medical Sciences (CAMS) and Linxian Cancer Hospital^28^. We included 19 tumour tissues and 19 germline tissues from this whole exome dataset.

For healthy control data, we created a healthy population from the combination of healthy reference data from two independent studies. These were 13 independent normal healthy HapMap exome data from Comino-Méndez I. *et al*.^29^ and 7 normal HapMap data from another independent study^27^.

The datasets and their details are:

1. Exome data from 11 familial non-BRCA1/BRCA2 breast cancer patients, having NCBI accession numbers ERR166303, ERR166304, ERR166307, ERR166308, ERR166310, ERR166312, ERR166315, ERR166330, ERR166333, ERR166335, ERR166336. In this paper they are referred to as BC1, BC2, BC3, BC4, BC5, BC6, BC7, BC8, BC9, BC10, and BC11 respectively.
2. Dataset containing exome data from 19 tumour tissues. These data have accession numbers SRR1044337 (ESCC-D1T), SRR1044333 (ESCC-D2T), SRR1044331 (ESCC-D3T), SRR1044329 (ESCC-D4T), SRR1044327 (ESCC-D5T), SRR1044325 (ESCC-D7T), SRR1044323 (ESCC-D8T), SRR1044321 (ESCC-D9T), SRR1044357 (ESCC-D10T), SRR1044355 (ESCC-D11T), SRR1044353 (ESCC-D12T), SRR1044351 (ESCC-D13T), SRR1044349 (ESCC-D14T), SRR1044347 (ESCC-D15T), SRR1044345 (ESCC-D16T), SRR1044343 (ESCC-D17T), SRR1044341 (ESCC-D18T), SRR1044339 (ESCC-D19T), SRR1044335 (ESCC-D20T). In this paper, these samples are labelled as T1, T2, T3, T4, T5, T7, T8, T9, T10, T11, T12, T13, T14, T15, T16, T17, T18, T19 and T20 respectively.
3. Dataset containing exome data from 19 matching distal germline tissues. Accession numbers for these samples are SRR1044338 (ESCC-D1N), SRR1044334 (ESCC-D2N), SRR1044332 (ESCC-D3N), SRR1044330 (ESCC-D4N), SRR1044328 (ESCC-D5N), SRR1044326 (ESCC-D7N), SRR1044324 (ESCC-D8N), SRR1044322 (ESCC-D9N), SRR1044358 (ESCC-D10N), SRR1044356 (ESCC-D11N), SRR1044354 (ESCC-D12N), SRR1044352 (ESCC-D13N), SRR1044350 (ESCC-D14N), SRR1044348 (ESCC-D15N), SRR1044346 (ESCC-D16N), SRR1044344 (ESCC-D17N), SRR1044342 (ESCC-D18N), SRR1044340 (ESCC-D19N), SRR1044336 (ESCC-D20N). In this paper, these samples are labelled as N1, N2, N3, N4, N5, N7, N8, N9, N10, N11, N12, N13, N14, N15, N16, N17, N18, N19 and N20 respectively.
4. Exome data from 13 HapMap healthy samples with accession numbers ERR031613, ERR031614, ERR031615, ERR031616, ERR031617, ERR031618, ERR031619, ERR031620, ERR031621, ERR031622, ERR031624, ERR031625, and ERR031626^29^. In this paper these samples are referred to as H1, H2, H3, H4, H5, H6, H7, H8, H9, H10, H11, H12, and H13 respectively.
5. Exome data of 7 HapMap normal samples^27^. These are labelled with ids ERR166316, ERR166318, ERR166320, ERR166323, ERR166325, ERR166326, and ERR166328. In this paper these samples are referred to as NA1, NA2, NA3, NA4, NA5, NA6, and NA7 respectively.

### SPKMG Computation

OncoTrack computed the SPKMG values as the Sequence Per Kilobase of exon, per Megabase of the mappable Genome. Intuitively, SPKMG is similar to RPKM^78^ or FPKM^79^, adapted for exome data with normalizations at the gene and genome level.

The formula for SPKMG is as follows:


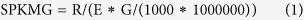


where,

R = Sequence reads aligned in exonic regions of a gene (after removal of PCR duplicates and low quality reads)

E = Length of genic region that includes only the exonic regions of this gene

G = Total number of reads that aligned to the reference genome

The SPKMG measures of breast cancer patients are presented as an example in Supplementary Table 1.

We plotted scatterplot matrices for the SPKMG values to examine the characteristics of the algorithm. The 11 breast cancer SPKMG values exhibited a linear correlation between any two samples. For the ESCC samples, the gene TTN had very high SPKMG measures (outlier) in both tumour and germline datasets and had to be removed for the analysis. The gene TTN has been previously mathematically associated with cancer^80^. Following removal of TTN, samples were seen to cluster into 2 distinct groups, with correlation within each group being more linear than between groups. The same patterns were observed for both germline and tumour samples. While the results for the germline samples became linear with near 1 correlations after removal of TTN, there was some deviation from this for tumour samples T5 and T13, due to additional outlier genes. The observed linear correlation indicates that the algorithm normalized the SPKMG values, and there are no differences (bias) between the samples or experiments^81^.

### Enrichment and Statistics of SPKMG

Algorithms such as the ChIP-Seq algorithm MACS assume DNA sequence alignment at genomic regions to follow a Poisson distribution^82^. Poisson distribution has also been used to model the number of reads obtained for each gene in RNA-Seq when only one technical replicate was present in the experiment^83^,^84^. However, negative binomial distribution is appropriate for modelling the additional variation observed between samples from biological replicates^85^. The Cuffdiff^86^ algorithm for differential expression for RNA-Seq assumes a negative binomial distribution. In case of SPKMG therefore, for a single sample (no replicate; N = 1), we assumed the distribution of sequence reads aligning to a genic region by chance will follow a Poisson distribution. However for the across sample comparative analysis, we used edgeR^30^,^31^,^85^, which assumes the underlying distribution to be negative binomial.

### Comparative Case/Control Analysis

In case/control analysis, we computed the comparative SPKMG for pairs of populations using edgeR. For this analysis, we used cancer data (11 breast cancer and 19+19 ESCC samples) as case and 20 healthy samples (13 H samples + 7 NA samples) as control. We then took these samples and examined how they clustered in MDS (Multi-dimensional scaling) plots using edgeR, along with hierarchical clustering for the samples.

We identified genes that were highly significant, and analysed them to identify their roles in cancer. We took 168 KEGG cancer related pathways from Broad Institute^33^ and used the XomPathways R script^34^, followed by network analysis, for the functional analysis of these genes. We also used DAVID, for further annotation.

### Control-free Analysis of Genes using Mutual Information

In control-free mutual information (MI) analysis, we used MI to discover the inherent relationship between genes, to elucidate the functional properties of SPKMG. MI is derived from information theory, which deals with the measure of uncertainty introduced by Shannon^87^. MI is defined as the relative entropy, of two random variables X and Y, given their joint and individual probabilities. If two components of a network interact closely, their MI will be high; if they do not interact, their MI will theoretically be zero.

MI for a pair of outcomes x and y of discrete random variables, X and Y, is defined as^88^,


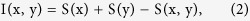


where S(t) is the entropy of an arbitrary variable *t*. Entropy for a discrete variable is defined as the average of the log probability of its states:


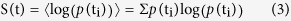


where p(t_i_) = Pr(t = t_i_) is the probability associated with each discrete state or value of the variable.

ARACNe^41^ uses MI to predict potential functional associations between genes by identifying statistical dependencies between gene expressions. We used parmigene^40^, an R statistical package with ARACNe to measure the MI between each pair of genes, to reconstruct the gene-gene interaction network with core genes. The BC (19358 × 11) SPKMG matrix of 11 breast cancer patients and the N and T (19358 × 19) ESCC SPKMG data were used to obtain 19358 × 19358 respective matrices of MI. We used appropriate thresholds for removing the weakest edge of each triple of nodes in parmigene and all gene-pairs with low MI coefficients to obtain the final adjacency matrices. The interaction networks constructed from these adjacency matrices were called the MI networks. The MI networks were then analysed for network properties. The results were validated by functional analysis of the shortlisted genes for biological processes and functional pathways using XomPathways and DAVID.

### Control-free Analysis using Pearson Correlation

We used Pearson correlation between tissues and genes in two cases. First, we computed the PC between the SPKMG values for germline and the matching tumour tissues for each patient across all genes. We then calculated and compared the gene-gene correlations for the germline and tumour population. For this, we created two 19358 × 19358 PC matrices for ESCC normal (germline) and matching ESCC tumour populations, by calculating the correlations between pairs of genes for all the samples in the population using the following equations.


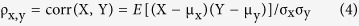



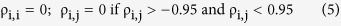



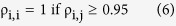



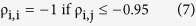


We quantized the PC matrices such that all PC above 0.95 were considered positive (PC = 1) and PC below −0.95 were considered negative (PC = −1), to construct adjacency matrices. We then constructed 3 interaction networks for each population, viz., positive correlation network, where all edges represent positive correlation, negative correlation network, where all edges represent negative correlation, and a complete correlation network, where edges represent non-zero correlations, after applying the thresholds. We studied the topological properties of these networks along with the functional properties of important genes and altered genes in these networks. Finally, we studied the link correlations between interaction networks created from germline and tumour populations.

### Comparison of SPKMG with CNV and SNP

We used GATK^25^ and XHMM^26^ protocols for calling SNVs and CNVs respectively. For the point mutation analysis, we used GATK haplotype caller on the recalibrated BAM files, followed by joint genotyping, variant quality score recalibration (VQSR) and variant annotation. We annotated the variants with the ClinVar database and 1000Genome allele frequencies and the SNPEff^89^,^90^ impact. Finally, the variants considered for analysis passed the VQSR filter, had minor allele frequency less than 0.01 in at least one 1000Genome population, and had a moderate/high impact as annotated by SNPEff.

For the CNV calls we followed the steps as suggested in the XHMM tutorial (https://atgu.mgh.harvard.edu/xhmm/tutorial.shtml). We calculated the read depths across the reference genome for all samples and removed the regions with outlier GC content. We then used a principal component analysis to remove the top 5 dimensions of variability which represent potential sources of noise in the data. Finally the CNVs were identified across all samples using the XHMM discover option. For this step, mean number of targets in CNV was set to 6, mean distance between targets within CNV was 70 KB, read depth distribution of deletion was specified as normal with mean −3 and variance 1. Duplication read depth distribution was specified as normal with mean 3 and variance 1. The Exome-wide CNV rate was given as 1e-08. The mean and standard deviation of the diploid z-score were set to 0 and 1 respectively.

## Additional Information

**How to cite this article**: Talukder, A. K. *et al*. Tracking Cancer Genetic Evolution using OncoTrack. *Sci. Rep.* **6**, 29647; doi: 10.1038/srep29647 (2016).

## Supplementary Material

Supplementary Information

Supplementary Table 1

Supplementary Table 2

Supplementary Table 3

Supplementary Table 4

Supplementary Table 5

Supplementary Table 6

Supplementary Table 7

## Figures and Tables

**Table 1:** Genes with highest statistical significance in Breast Cancer case/control comparative analysis.

Gene	Description	P-value	Number of Unique Samples (COSMIC)	COSMIC Studies	COSMIC References
TCEAL5	transcription elongation factor A (SII)-like 5	3.26E-67	28,859	13	14
PRB4	proline-rich protein BstNI subfamily 4	1.44E-34	28,981	24	32
PRB2	proline-rich protein BstNI subfamily 2	6.63E-33	28,772	24	26
SPRR2D	small proline-rich protein 2D	1.83E-27	28,792	8	8
FLG	Filaggrin	6.01E-27	28,983	43	92
FAM47C	family with sequence similarity 47, member C	1.23E-26	28,796	35	52
MKI67	marker of proliferation Ki-67	1.26E-26	28,903	38	77
OTOP1	Otopetrin 1	4.45E-25	28,815	38	42
AHNAK	AHNAK nucleoprotein	5.35E-25	29,029	41	83
OR10A2	olfactory receptor, family 10, subfamily A, member 2	2.01E-24	28,838	19	23
MYH1	myosin, heavy chain 1, skeletal muscle, adult	2.83E-24	29,073	41	81
CSH2	chorionic somatomammotropin hormone 2	6.53E-24	28,814	15	9
SPRR2B	small proline-rich protein 2B	7.71E-24	28792	7	9
GH2	growth hormone 2	3.07E-23	28,815	21	19
CSH1	chorionic somatomammotropin hormone 1 (placental lactogen)	4.41E-23	24,523	20	19
TCEAL6	transcription elongation factor A (SII)-like 6	4.88E-23	28,814	20	18
KIR3DL1	killer cell immunoglobulin-like receptor, three domains, long cytoplasmic tail, 1	8.85E-23	28,831	6	11
MAGEC1	melanoma antigen family C1	4.17E-22	28,937	40	72
KRT33A	keratin 33A, type I	5.09E-22	28,816	28	25
OR10G8	olfactory receptor, family 10, subfamily G, member 8	2.18E-21	28,816	26	33
RFPL1	ret finger protein-like 1	2.69E-21	28,815	24	18
MAGEA3	melanoma antigen family A3	2.72E-21	28,814	19	16
KRT6B	keratin 6B, type II	3.18E-21	28,815	31	35
PDE4DIP	phosphodiesterase 4D interacting protein	4.75E-21	25,256	39	67
SPRR2F	small proline-rich protein 2F	6.69E-21	28,792	12	10
KRTAP21-1	keratin associated protein 21-1	7.27E-21	28,855	9	12
IFNA21	interferon, alpha 21	7.94E-21	24,540	13	15
OR4A47	olfactory receptor, family 4, subfamily A, member 47	7.99E-21	28,815	23	32

All 28 most significant genes have been discovered in multiple cancer studies.
