# Supplementary material for: Tracking Cancer Genetic Evolution using OncoTrack
Source: Sci Rep. 2016 Jul 14;6:29647. doi: 10.1038/srep29647 (PMC4944131; doi:10.1038/srep29647)
Supplement: Supplementary Information [file srep29647-s1.pdf]

Tracking Cancer Genetic Evolution using OncoTrack

Asoke K. Talukder[1]*, Mahima Agarwal[2], Kenneth H. Buetow[3], Patrice P. Denèfle[4]

Supplementary Information

Supplementary Information 1: Bibliomic Analysis of Genes from Breast Cancer

Comparative Analysis of SPKMG

We got 28 genes with adjusted p-values ranging from 6.3E-063 to 5.5E-018 from the breast cancer case/control comparative analysis. These genes with their individual p-values are, TCEAL5 (6.32E-63), PRB4 (1.39E-30), PRB2 (4.28E-29), SPRR2D (8.86E-24), FLG (2.33E-23), FAM47C (3.48E-23), MKI67 (3.48E-23), OTOP1 (1.08E-021), AHNAK (1.15E-021), OR10A2 (3.9E-21), MYH1 (4.97E-21), CSH2 (1.05E-20), SPRR2B (1.14E-20), GH2 (4.25E-20), CSH1 (5.69E-20), TCEAL6 (5.90E-20), KIR3DL1 (1.01E-19), MAGEC1 (4.49E-19), KRT33A (5.16E-19), OR10G8 (2.11E-18), RFPL1 (2.39E-18), MAGEA3 (2.39E-18), KRT6B (2.67E-18), PDE4DIP (3.83E-18), SPRR2F (5.18E-18), KRTAP21-1 (5.41E-18), IFNA21 (5.53E-18), and OR4A47 (5.53E-18).

Following are some of the other studies on these 28 genes,

1. TCEAL5 (Melanoma[91], Pancreatic cancer[92]).
2. PRB4 (Esophageal adenocarcinoma[93], Myeloproliferative neoplasm[94], Melanoma[91], Head and neck squamous cell carcinoma[95])
3. PRB2 (Melanoma[91], Pancreatic cancer[92,96]).
4. SPRR2D (Esophageal adenocarcinoma[93], Melanoma[91], Pancreatic cancer[92]).
5. FLG (Esophageal adenocarcinoma[93], Pancreatic cancer[97,92], Myeloproliferative neoplasm[94], Melanoma[91,98], Colorectal carcinoma[99], Head and neck cancer[98], Bronchus and Lung cancer[98], Urinary cancer[98], Nonmelanoma skin cancer[98], Prostate cancer[98,100], Breast

Cancer[98], Colorectal cancer[98]).

6. FAM47C (Esophageal adenocarcinoma[93], Myeloproliferative neoplasm[94], Melanoma[91], Pancreatic cancer[96], Prostate cancer[100]).
7. MKI67 (Esophageal adenocarcinoma[93], Melanoma[91], Colorectal carcinoma[99,101], Pancreatic cancer[91], Prostate cancer[102,94]).
8. OTOP1 (Esophageal adenocarcinoma[93], Pancreatic cancer[97], Melanoma[91], Lung cancer[103], Prostate cancer[100]).
9. AHNAK (Esophageal adenocarcinoma[93], Myeloproliferative neoplasm[94], Melanoma[91], Head and neck squamous cell carcinoma[95], Pancreatic cancer[92], Prostate cancer[100]).
10. OR10A2 (Melanoma[91]).
11. MYH1 (Esophageal adenocarcinoma[93], Pancreatic cancer[97], Myeloproliferative neoplasm[94], Melanoma[91], Prostate cancer[100]).
12. CSH2 (Esophageal adenocarcinoma[93], Melanoma[91]).
13. SPRR2B (Esophageal adenocarcinoma[93], Melanoma[91]).
14. GH2 (Esophageal adenocarcinoma[93], Melanoma[91], Pancreatic cancer[92]).
15. CSH1 (Esophageal adenocarcinoma[93], Melanoma[91], Prostate cancer[100]).
16. TCEAL6 (Esophageal adenocarcinoma[93], Melanoma[91], Pancreatic cancer[92], Prostate cancer[100]).
17. KIR3DL1 (Melanoma[91], Colorectal Cancer[104,105], Pancreatic Cancer[105], Gastric Cancer[105]).
18. MAGEC1 (Esophageal adenocarcinoma[93], Melanoma[91,106], Head and neck squamous cell carcinoma[95], Mantle cell lymphoma[107], Pancreatic cancer[96]).
19. KRT33A (Esophageal adenocarcinoma[93], Melanoma[91], Prostate cancer[100]).
20. OR10G8 (Esophageal adenocarcinoma[93], Melanoma[91], Prostate cancer[100]).
21. RFPL1 (Melanoma[91], Prostate cancer[100], Colorectal cancer[99]).
22. MAGEA3 (Melanoma[91], Colorectal cancer[99], Gingivo-buccal oral squamous cell carcinoma[108], Neuroblastoma[110]).

23. KRT6B (Melanoma[91], Lung adenocarcinoma[109], Neuroblastoma[110]).
24. PDE4DIP (Melanoma[91], Colorectal cancer[99], Lung adenocarcinoma[109], Gingivo-buccal oral squamous cell carcinoma[108], Neuroblastoma[110], Chondrosarcoma[111]).
25. SPRR2F (Melanoma[91], Colorectal cancer[99]).
26. KRTAP21-1 (Melanoma[91], Colorectal cancer[99], Lung adenocarcinoma[109]).
27. IFNA21 (Melanoma[91], Colorectal cancer[99], Lung adenocarcinoma[109], Pancreatic cancer[92]).
28. OR4A47 (Melanoma[91], Colorectal cancer[99], Lung adenocarcinoma[109], Neuroblastoma[110]).

References:

91. Berger, M.F. *et al.* Melanoma genome sequencing reveals frequent PREX2 mutations. *Nature* **485**, 502-506 (2012).
92. Jones, S. *et al.* Core Signaling Pathways in Human Pancreatic Cancers Revealed by Global Genomic Analyses. *Science (New York, NY).* **321**, 1801-1806 (2008).
93. Dulak, A.M. *et al.* Exome and whole genome sequencing of esophageal adenocarcinoma identifies recurrent driver events and mutational complexity. *Nature genetics* **45**, 10 (2013)
94. Nangalia, J. *et al.* Somatic CALR Mutations in Myeloproliferative Neoplasms with Nonmutated JAK2. *N Engl J Med.* **369**, 2391-2405 (2013).
95. Stransky, N. *et al.* The Mutational Landscape of Head and Neck Squamous Cell Carcinoma. *Science (New York, NY).* **333**, 1157-1160 (2011)
96. Wu, J. *et al.* Whole-exome sequencing of neoplastic cysts of the pancreas reveals recurrent mutations in components of ubiquitin-dependent pathways. *Proc Natl Acad Sci U S A.* **108**, 21188-21193 (2011).
97. Jiao, Y. *et al.* Whole Exome Sequencing of Pancreatic Neoplasms with Acinar Differentiation. *J Pathol.* **232**, 428-435 (2014).
98. Skaaby, T. *et al.* Filaggrin loss-of-function mutations and incident cancer: a population-based study. *Br J Dermatol* **171**, 1407-1414 (2014).

99. The Cancer Genome Atlas Network. Comprehensive Molecular Characterization of Human Colon and Rectal Cancer. *Nature*. **487**, 330-337 (2012).

100. Barbieri, C.E. *et al.* Exome sequencing identifies recurrent SPOP, FOXA1 and MED12 mutations in prostate cancer. *Nature genetics*. **44**, 685-689 (2012).

101. Bass, A.J. *et al.* Genomic sequencing of colorectal adenocarcinomas identifies a recurrent VTI1A-TCF7L2 fusion. *Nat Genet.* **43**, 964-968 (2011).

102. Rizzardi, A.E. *et al.* Evaluation of protein biomarkers of prostate cancer aggressiveness. *BMC Cancer* **14**, 244 (2014).

103. Liu, J. *et al.* Genome and transcriptome sequencing of lung cancers reveal diverse mutational and splicing events. *Genome Res* **22**, 2315-2327 (2012).

104. Kim, H.J. *et al.* HLA-Cw polypmorphism and killer cell immunoglobulin-like receptor (KIR) gene analysis in Korean colorectal cancer patients. *Int J Surg.* **12**, 815-820 (2014).

105. Peng, Y.P. *et al.* Comprehensive analysis of the percentage of surface receptors and cytotoxic granules positive natural killer cells in patients with pancreatic cancer, gastric cancer, and colorectal cancer. *J Transl Med* **11**, 262; *DOI:10.1186/1479-5876-11-262* (2013).

106. Krauthammer, M. *et al.* Exome sequencing identifies recurrent somatic RAC1 mutations in melanoma. *Nat Genet.* **44**, 1006-1014 (2012).

107. Beà, S. *et al.* Landscape of somatic mutations and clonal evolution in mantle cell lymphoma. *Proc Natl Acad Sci U S A.* **110**, 18250-18255 (2013).

108. India Project Team of the International Cancer Genome Consortium. Mutational landscape of gingivo-buccal oral squamous cell carcinoma reveals new recurrently-mutated genes and molecular subgroups. *Nat Commun.* **4**, 2873; *DOI:10.1038/ncomms3873* (2013).

109. Imielinski, M. *et al.* Mapping the hallmarks of lung adenocarcinoma with massively parallel sequencing. *Cell* **150**, 1107-1120 (2012).

110. Pugh, T.J. *et al.* The genetic landscape of high-risk neuroblastoma. Nat Genet. **45**, 279-284 (2013).

111. Tarper P.S. *et al.* Frequent mutation of the major cartilage collagen gene COL2A1 in chondrosarcoma. *Nat Genet.* **45**, 923-926 (2013).

Supplementary Information 2: Functional and Centrality Analysis of Breast Cancer Mutual Information Network

Functional enrichment analysis for 11 Breast cancer MI network key genes, along adjusted enrichment p-values returned the biological processes 'cell-cell adhesion' (adjusted p-value 6.5E-3), 'cell adhesion' (adjusted p-value: 5.3E-3), and 'biological adhesion' (adjusted p-value: 3.6E-3).

Centrality Analysis

Centrality analysis of the breast cancer MI network was carried out using different centrality measures. The key genes identified from this analysis are:

1. Degree centrality: CSMD3, DCC, FLRT3, DIAPH3, SPAM1, ARHGAP24, DACH1, LIPI, BCHE, GLRB
2. Closeness centrality: DMD, ANO3, CXORF22, CSMD3, LRP1B, BRWD3, C12ORF50, GALNTL6, ZNF804A, C6ORF118
3. Betweenness centrality: CXORF22, C12ORF50, DMD, DSC2, CSMD3, LRP1B, ANO3, TRHDE, DCC, GCNT4
4. Eigenvector centrality: FLRT3, SPAM1, POF1B, GLRB, BCHE, OR13C8, PACRGL, HAS2, XIRP2, SLITRK6

The above list of 40 genes has a few common genes. After removal of these common genes, there are 28 unique genes that emerge as central from the different centrality analyses. These 28 genes are CSMD3, DCC, FLRT3, DIAPH3, SPAM1, ARHGAP24, DACH1, LIPI, BCHE, GLRB, DMD, ANO3, CXORF22, LRP1B, BRWD3, C12ORF50, GALNTL6, ZNF804A, C6ORF118, DSC2, TRHDE, GCNT4, POF1B, OR13C8, PACRGL, HAS2, XIRP2, SLITRK6. Of these, genes such as DCC, DIAPH3, ARHGAP24, BCHE, DMD, LRP1B, BRWD3, DSC2, and HAS2 are associated with metastasis and tumorigenesis[112,113,114,115,116, 117, 118, 119, 120].

References:

112. Krimpenfort, P. e*t al.* Deleted in colorectal carcinoma suppresses metastasis in p53-deficient mammary tumours. *Nature.* **482**, 538-41 (2012).

113. Di Vizio, D. e*t al.* Oncosome formation in prostate cancer: association with a region of frequent chromosomal deletion in metastatic disease. *Cancer Res.* **69**, 5601-5609 (2009).

114. Feng, M. *et al.* RASAL2 activates RAC1 to promote triple-negative breast cancer progression. *J Clin Invest.* **124**, 5291-5304 (2014)

115. Battisti, V. *et al.* Cholinesterase activities and biochemical determinations in patients with prostate cancer: influence of Gleason score, treatment and bone metastasis. *Biomed Pharmacother.* **66**, 249-255 (2012).

116. Pantaleo, M.A. *et al.* Dystrophin deregulation is associated with tumor progression in KIT/PDGFRA mutant gastrointestinal stromal tumors. *Clin Sarcoma Res.* **4**, 9; *DOI:10.1186/2045-3329-4-9* (2014).

117. Craig, D.W. *et al.* Genome and transcriptome sequencing in prospective metastatic triple-negative breast cancer uncovers therapeutic vulnerabilities. *Mol Cancer Ther*. **12**, 104-116 (2013).

118. Eui Jin Suh *et al.* Comparative profiling of plasma proteome from breast cancer patients reveals thrombospondin-1 and BRWD3 as serological biomarkers. *Exp Mol Med.* **44**, 36-44 (2012).

119. Fang, W.K. *et al.* Down-regulated desmocollin-2 promotes cell aggressiveness through redistributing adherens junctions and activating beta-catenin signalling in oesophageal squamous cell carcinoma. *J Pathol.* **231**, 257-270 (2013).

120. Wu, M. e*t al.* A novel role of low molecular weight hyaluronan in breast cancer metastasis. *FASEB J.* **29**, 1290-1298 (2015).

Supplementary Information 3: Common SNVs and InDels identified across all ESCC samples

The rsIDs of the 20 common mutations across tumor and germline ESCC samples are given below. Some mutations are associated with more than 1 rsID.

1. rs72559129, rs112595382
2. rs113322110; rs3214485
3. rs3827760
4. rs113934564, rs66744502
5. rs55765823
6. rs76159126, rs5872508
7. rs4535533
8. rs11402364, rs75544239
9. rs67335052, rs36008849
10. rs33914855; rs11411516
11. rs67530050, rs113178278
12. rs10897158
13. rs139455912, rs11455434
14. rs5795166, rs33936067
15. rs55912941, rs150851358
16. rs936212
17. rs3803354
18. rs1426654
19. rs57321480, rs75244038
20. rs56070390
